# Cross-Reaction between the Crude Hydatid Cyst Fluid Antigens of Human and Animals Origin in Response to Human IgG Class and Subclasses

**DOI:** 10.1155/2012/947948

**Published:** 2012-03-13

**Authors:** Afra Khosravi, Sobhan Ghafourian, Morteza Shamsi, Nourkhoda Sadeghifard, Abbas Maleki, Ebrahim Babaahmadi

**Affiliations:** ^1^Immunology Department, Faculty of Medicine, Ilam University of Medical Sciences, Ilam 6931994585, Iran; ^2^Clinical Microbiology Research Center, Ilam University of Medical Sciences Iran, Ilam 6931994585, Iran; ^3^Parasitology Department, Faculty of Veterinary Sciences, Ilam University, Ilam 6931994585, Iran

## Abstract

The current work aimed to evaluate the cross-reactivity of human immune sera against crude hydatid fluid antigens of sheep, human, mouse, cattle, as well as B fraction of cystic fluid antigen. 30 balb/c mice were infected with sheep hydatid cyct fluid antigen containing protoscolex after the viability of these protoscolices was assessed. ANOVA was used to test the difference of themean of optical density (OD) values among case and control groups. The highest human IgG class antibody was against antigen B (0.93) and the lowest against cattle HCF antigen (0.32). The differences between responses to these antigens were statistically significant (*P* < 0.001). The sensitivity and specificity of ELISA test used for evaluating the responses of human total IgG to different hydatid cyst fluid (HCF) antigens among the case and control groups were 100 and 95.8%, respectively. Cross-reaction of human IgG class and subclasses responses was found almost for all the antigens with the best reaction against human and cattle (HCF) antigens and antigen B using a ratio of mean OD value to each antigen divided by the cut-off point value for the same antigen. Human sera showed a considerable cross-reactivity against all antigens by using ELISA.

## 1. Introduction

Hydatidosis is a chronic, cyst-forming, parasitic disease of human and domestic or wild animals. It is caused by infection with the larval stages of dog/fox tapeworms (cestodes) belonging to the genus *Echinococcus *(family Taeniidae), which is also referred to as Echinococcosis [1]. The two major species of medical and public health importance are *Echinococcus granulosus *and *Echinococcus multilocularis*, which cause cystic Echinococcosis (CE) and alveolar Echinococcosis (AE), respectively. Human hydatid cyst is the most common presentation and probably accounts for more than 95% of the estimated 3 million global cases. Human AE causes approximately 0.3–0.5 million cases (all in the Northern Hemisphere) annually [1]. As these parasites are complex multicellular pathogens that are able to modulate antiparasite immune responses and persist and flourish in their mammalian hosts, understanding how the immune system deals with these parasites is a major challenge [1]. Though recent application of modern molecular and immunological approaches has elucidated some insights on the nature of immune responses generated during the course of hydatid infection, many aspects of the *Echinococcus *host interplay have remained unexplored yet [1].

 If the hydatid cyst is formed in vital organs like brain and heart, the risks of the disease will be more serious. This disease is widespread in most countries in the world especially in sheep raising areas. The disease causes so many economic and health problems annually [2, 3]. Higher prevalence of hydatidosis is reported from tropical and subtropical areas such as Mediterranean region, Europe, central Asia, Middle East, Far East, Russia, Australia, New Zealand, America, and Africa. This disease has been reported from all the provinces of Iran, with the highest prevalence in Khorasan (4.45 per 100,000 populations) and the lower prevalence in Hormozgan (0.1 in 100,000 populations), while the overall prevalence of the disease for the whole country is about 1.2 in 100,000 population. Iran is a hyper-endemic area for infection by *Echinococcus granulosus* [4].

Rural populations, who are in close contact with domestic and wild animals such as dogs and canine, especially those living in undeveloped countries, are more in risk of infection. Approximately, 60% of hydatidosis cases are asymptomatic, so that the disease may remain in the human body for a period of 20 years. As parasite can habituate in different organs like lung, heart, brain, liver, spleen, and spinal cord, diagnosing the disease is so difficult and usually based on paraclinical methods like serology [5]. Since surgery has been considered the only treatment available for such a disease, definitive and immediate diagnosis of hydatidosis is vital for the patients [6]. The use of crude hydatid cyst fluid (HCF) antigen for the diagnosis of cystic hydatid (CH) is a method which along with immediate serological investigation can be helpful and effective in rapid treatment of the disease [7]. It seems that the antigen of human origin can be more useful for the diagnosis of hydatid cyst, as it has reportedly been confirmed that the HCF from a human CE patient shows a relatively stronger positive reaction [8]. Preparing human HCF antigen is too difficult and not applicable everywhere and all the time. If serological diagnosis is based on the use of some highly valid and reliable animal antigen instead of human antigen, designing the diagnostic kit with such an antigen can be a simple, nonexpensive, and available method. Most the serological tests used in diagnosing the hydatid cyst have their own problems such as limited availability, different sensitivity, and specificity or difficulty in their preparation. Some of these tests need several specific techniques, equipment, and experienced staff.

Of the serological tests for detecting anti-*Echinococcus *serum antibodies, the enzyme-linked immunosorbent assay, the indirect hem agglutination antibody test (IHAT), and the latex agglutination test (LAT) are commonly used in laboratories [8], while the immunofluorescence antibody test (IFAT), immunoelectrophoresis (IEP), and some other tests are employed less frequently. In many countries, the materials, reagents, and equipment to perform the IgG-ELISA are easily available, and this technique is probably the best choice for use in immunodiagnosis for human CE. However, there is still no standard, highly sensitive, and specific serological test for antibody detection in cases of human CE [8]. Therefore, for clinical practice, it should be noted that the results of serological tests depend on several factors, such as antigen quality, test system, organ site, the number of hydatid cysts, and individual variability of immune responses. The IgG-ELISA is one of the most sensitive tests available now [9]. The IFAT has sensitivity similar to that of the ELISA-IgG. Because of the variable sensitivities of the various tests, many laboratories employ at least two different primary tests for routine diagnosis of CE which usually increases the sensitivity [10, 11]. Different studies have reported a sensitivity range of 60–90% for ELISA and a specificity range of 75–90% [12]. Usually the range varies based on antigen type, methodology, the geographical region in which the test is performed, and also the endemic region of the disease. Sometimes the cross- reaction occurs in serologic tests and reduces the accuracy of the diagnosis, which can be confirmed best by performing the immune-blotting test. On the other hand, antigens have different fractions; for example, antigen B has 8, 16, 24, and 38 KD with different sensitivities in diagnosis of the disease [13, 14]. Also, these fractions have different sensitivities in each animal as well; therefore, finding an antigen with a fraction whose sensitivity and specificity are high in response to the sera of man or other animals can be a considerable progress in developing a reliable and nonexpensive method for diagnosis of HC.

**Figure 1:**
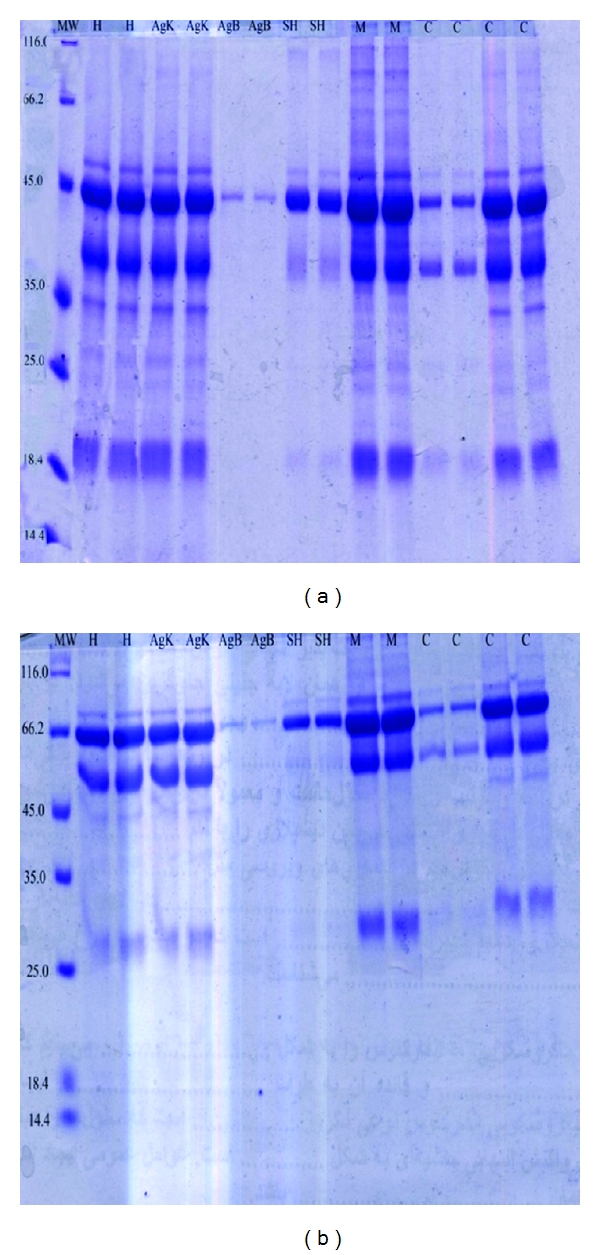
SDS PAGE showing different fractions of human and animal HCFs. MW: molecular weight; H: human; B: antigen B; AgK: antigen hydatid kit; SH: sheep; M: mice; C: cattle.

In order to access such a goal, the first step is to prepare an appropriate crude antigen of parasite from different sources (human or animals) which can be recognized by most the sera of man and animal. The purification and evaluation steps of antigens with good quality and quantity are the next steps. However, it is possible that an appropriate crud antigen in some cases indicates an acceptable efficiency in serological experiments for preparing certain antigens. Which antigen can respond to human antibodies more strongly and effectively than the others? Which fraction is more immunologic? Which immunoglobulin class or subclass has the best reaction and is preferred for these responses? This study was designed to give some clearer answers to such questions based on valid observations applied to the conditions of localized antigens (Figure 1).

## 2. Material and Methods

This is an analytical case-control study using human and animal crude HCF as the source of antigen for performing ELISA, Western blotting, and immunization of mice. Sample sera used in present work were collected from patients who had recently undergone hydatid surgeries in hospitals of Tehran, Hamadan, and Ilam cities as human case group together with some human or animal sera with no history of hydatidosis with negative HC by using ELISA and IFAT as control group. Briefly, 30 balb/c mice were infected with fluid containing protoscolex of sheep HC after their viability was assessed by Eozin method. The required antigen was extracted and prepared from naturally infected human, sheep, and cattle along with mice hydatid fluid cysts.

### 2.1. Statistical Analysis

Sample size was calculated with using *n* = *λ*/Δ formula, that *λ* = 15.4 (*λ*, for *α* = 5%, *β* = 10%, and *k* = 5 groups, is 15.4), $\Delta =\left(\sum_{i=1}^{5}{\left(\mu_{i}-\overset{®}{\mu }\right)}^{2}\right)/\sigma^{2}$, and *μ*_*i*_ were mean OD in the five groups. ANOVA was used to test difference mean of OD among groups. Test of homogeneity of variances was done by Levens statistics. Tukey test was used in the post hoc analysis. *P*-values less than 5% were considered as statistically significant.

## 3. Results

### 3.1. Results of HC Formation in Mice Experimentally Infected with Protoscolices

All the experimentally infected mice showed hydatid cysts 6–9 months after receiving protoscolices from sheep origin. In mouse, both the amount and rate of HC formation were the highest in liver and abdomen, respectively. While 10 out of 30 balb/c mice were infected after 6–9 months, 30 out of 30 ICR mice showed HC during the same period.

### 3.2. ELISA Results Using Human Sera

For all the 30 human sera in either the case or the control groups, the total IgG nd IgG subclass antibodies (IgG2, IgG3, and IgG4) against HCFs antigens of human, mouse, sheep, and cattle origin together with antigen B were measured using ELISA. The cut-off values for each antibody were calculated separately together with sensitivity and specificity which was calculated for each antigen individually (Tables 1, 2, 3, 4, 5, and 6).

When HCFs from the four different host origins (cattle, mice, sheep, and human) were used in ELISA, similar results with all 30 human sera were observed, so that IgG4 had the highest mean against most antigens (excepted for mice HCF), while IgG total stood at the second place. Cross-reaction of human sera was found against all these antigens with the highest mean optical density (OD) value against human HCF and antigen B (Tables 1–6). When analysis was applied for IgG class and subclasses against human, mice, cattle HCFs, and antigen B in control group, the IgG4 had the highest mean OD value. Statistically, the difference between the OD values of all antibodies against these antigens was significant but the OD values of all antibodies to each individual antigen were less than those of the cut-off: hence, the results proved negative and there was no need for further analysis (Table 2). On the other hand, when ELISA was carried out to examine the IgG class and subclass analysis against human, mice, and sheep HCF antigens along with antigen B in the case group, the results for each antigen were different from those of the others. IgG3 had the lowest mean OD values to human HCF antigens. As the mean OD values for all antibodies in response to human antigen were higher than those of the cut-off, all the human sera samples are regarded as positive, indicating a cross-reaction of human antibodies to animals and human HCFs. ANOVA analysis showed that the difference between the mean OD values of human antibodies against hydatid cyst crude antigen from different origin was statistically significant (Table 8) (*P* < 0.01). In other words, human humoral immune responses against different hydatid cyst crude antigens are totally different. IgG4 had the highest reaction among the other IgG subclasses. At the same time, another parameter was calculated as “OD ratio” by dividing mean OD value of each antigen to its cut-off. Though cross-reaction of human IgG response was observed against all the antigens using OD ratio, the strength of this response was different and this difference was statistically significant using ANOVA (*P* < 0.01). The sensitivity of ELISA for human IgG class against human crude HC antigen was 100%, while its specificity was 95.8%.

### 3.3. ELISA Results for Human HCF

Human IgG4 had the highest OD value (0.59) and IgG3 the lowest (0.16) against human crude fluid antigens of hydatid cyst (Table 1). This was the second highest human IgG response after antigen B (Table 7). Regarding IgE response to different HCF antigens, the highest OD value was observed against human HCF and the lowest against mice HCF antigen (Table 10). Generally speaking, IgG4, IgG, and IgE were the highest antibody responses of human sample sera against HCF of human origin. Also, amongst human antibody responses, the IgG4 took the first place with OD ratio of 10.5, while the IgE took the second and IgG class at the third place with OD ratio of 8.2 and 7.4, respectively (Table 11).

### 3.4. ELISA Results for Sheep HCF

IgG4 had the highest mean OD against sheep HCF antigen, while IgG2 the lowest (Table 10). The reactivity of human IgG class and subclasses to sheep HCF antigen was high so that the OD values of all the antibodies were above the cut-off. The pattern of human antibody response to sheep HCF was similar to that of human HCF, hence, similar to cross-reactivity of human antibody to these two antigen sources. The antibody response to human HCF was significantly stronger than that to sheep HCF. In order to analyze the strength of antibody responses, the OD ratios of all the antibodies were computed. The higher ratio was found for IgG4 at the first place and IgG total at the second place (Table 11). ANOVA showed a significant difference between the mean OD of antibody responses to this antigen (*P* < 0.01).

### 3.5. ELISA Results for Mice HCF

For this antigen again the higher mean OD value was found for human IgG, while the higher OD ratio was seen for human IgG4 (Tables 10-11). In other words, human IgG4 and IgG were the most reactive antibody to mice HCF, respectively, using ELISA, a pattern similar to that of human and sheep HCF.

### 3.6. ELISA Results for Cattle HCF

A similar picture was drawn for human antibody against cattle HCF with the higher mean OD value and also stronger antibody response (OD ratio) for IgG4 and IgG, respectively.

### 3.7. ELISA Results for B Antigen

IgG4 and surprisingly IgG2 were the strongest responses of human antibody against antigen B, while other antibody responses were high too. It is worth to say that the pattern was similar to the above mentioned antigens showing that human sera can react with the HCF antigens of human and animals at a similar way even with stronger response against some animal antigens bearing in mind the hypothesis of this study that there is a cross-reaction of antibody to different antigens. 

### 3.8. Results of Sodium Dodecyl Sulfate Polyacrylamide Gel Electrophoresis (SDS-PAGE)

Similar patterns of antigen fractions were seen in SDS PAGE for almost all the antigens studied in present work. This similarity was more remarkable for human, mice, and cattle.

### 3.9. Different HCFs in Response to Human IgG Class

ANOVA and post hoc analysis test for human HCF antigen with antigens from the other animals (mice, sheep, cattle, and antigen B) showed a significant difference of mean OD in response to human IgG class response (*P* < 0.01) (Table 9). The same analysis for sheep HCFs with other HCFs was also assessed (Table 10) resulting in a significant difference in mean OD between the sheep HCF and the human one (*P* < 0.01), while this analysis showed no statistically significant difference in mean OD values for sheep antigen with mice and cattle ones. 

Finally, when the analysis was carried out for the difference mean OD to mice HCF antigens with that of other resources, the difference was not significant for mice HCF with sheep and cattle but it was significant for the mice HCF with that of human (*P* < 0.01).

## 4. Discussion and Conclusion

 IgG4 subclass showed the highest mean OD values amongst other human IgG subclasses against human HCF which is in agreement with the results of studies performed by Siracusano et al. [15], Khabiri et al. [16], Wen and Craig [17], Dreweck et al. [18], and Grimm et al. [19]. The mean OD values of human IgG4 against sheep and mice HCF were also the highest compared to other IgG subclasses. These are the evidences indicating that IgG class and IgG4 subclass had the highest responses against all HCF antigens investigated at present study. As it was demonstrated in our study, this IgG subclass had the highest mean OD values against cattle HCF antigen and antigen B too, which is in line with the results of studies carried out by Siracusano et al. [15] and Wen and Craig [17]. As it was previously mentioned, the aim of the current study was to find the best antibody which is reactive against all the antigens and also the most reactable antigen against the human sera antibodies. In other words, finding an antigen in human, cattle, mice, and sheep which has the best cross-reaction in response to human antibody was aimed in this study. It seems that the human HCF and antigen B are at the top of HCF antigens for this purpose according to the mean OD values obtained. In order to make a better analysis, the ratio of mean OD values to that of the cut-off for each antigen was calculated as the positivity of each antigen usually was based on this ratio. This ratio was primarily obtained for human IgG4 against each antigen. The ratio of human IgG4 against human HCF was 10.5. In other words, the mean OD value of human IgG4 against human HCF was 10.5 times of its cut-off values. Cattle (10.2), antigen B (9), and mice (7.25) were at the next places, respectively. There were significant differences in the ratios of IgG response to different antigens (*P* < 0.01). As a conclusion, it can be said that human HCF, antigen B, and cattle HCF had the best cross-reaction by using human IgG4 as the best IgG subclass response for this purpose. It can also be seen from the results of human IgG class and IgG subclass analysis that the difference between the HCF antigens was statistically significant (*P* < 0.01) using ANOVA. These data imply that some antigens are stronger than others in raising human sera reaction and also some IgG subclasses are preferred over others in diagnosing HCF antigens of different origins, which was confirmed by Sbihi et al. [20] and Grimm et al. [19] too. Though the highest ratio of IgG response compared to its cut-off was higher for human HCF, there is a considerable ratio for mice HCF too. Mice HCF can be accessed more easily and applied for diagnosis of hydatid cyst effectively. 

**Figure 2:**
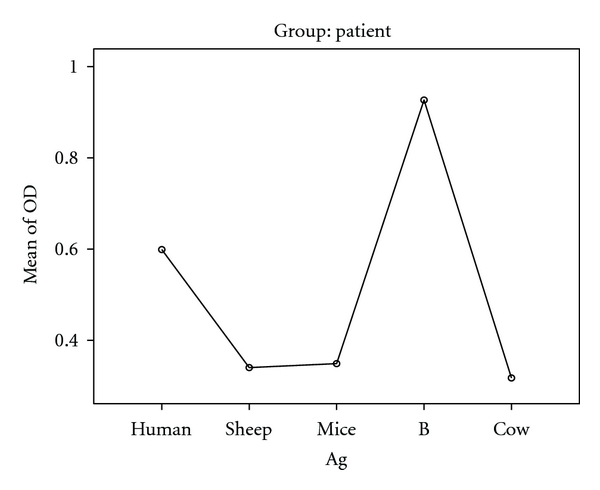
The mean OD values of total Human IgG response against mice, cattle, human, and sheep HCF along with antigen B.

 Generally speaking, when ELISA was carried out using human IgG class, the highest mean OD value was found against antigen B at the first place, while the human HCF took the next place (Figure 2). Cross-reaction strongly exists between HCF antigens of human and animals origin of which human and mice antigen are remarkable.

## 5. Limitations

In clinical practice tests for detecting specific serum antibodies are of particular importance in the diagnosis of CE, whereas detection of circulating antigens is less relevant. Even if highly sensitive tests are used, such as the IgG-ELISA, antibodies may not be detectable in a certain proportion of patients with Echinococcosis (false-negative results). Cysts in the brain or eye and calcified cysts often induce low or no antibody titres. Antibody response may also be low in certain human population groups and in young children. False positive results may also occur, especially in patients with other helminthic diseases [21].

## Figures and Tables

**Table 1:** The mean OD values of IgG class and subclass responses of 30 naturally infected hyadid patients against human cystic crude fluid antigens.

OD	Mean	Std. deviation	Minimum	Maximum	Number	Range	Cut-off
IgG	0.59	0.02	0.51	0.68	30	0.08	0.08
IgG2	0.34	0.04	0.22	0.41	30	0.19	0.12
IgG3	0.16	0.04	0.12	0.38	30	0.26	0.11
IgG4	1.58	0.18	1.31	1.99	30	0.68	0.15
IgE	0.49	0.17	0.15	0.76	30	0.62	0.06

(*P* = 0.01, *F* = 725.38).

**Table 2:** The The mean OD values of IgG class and subclass responses of 30 healthy individuals against human cystic crude fluid antigens.

OD	Mean	Std. deviation	Minimum	Maximum	Number	Range	Cut-off
IgG	0.04	0.02	0.01	0.11	30	0.1	0.08
IgG2	0.04	0.04	0	0.13	30	0.13	0.12
IgG3	0.05	0.03	0.01	0.12	30	0.12	0.11
IgG4	0.09	0.04	0	0.15	30	0.15	0.15
IgE	0.02	0.04	0	.06	30	.06	.06

*P* = 0.01; *F *= 15.98.

**Table 3:** The mean OD values of IgG class and subclass responses of 30 naturally infected hyadid patients against sheep cystic crude fluid antigens.

OD	Mean	Std. deviation	Minimum	Maximum	Number	Range	Cut-off
IgG	0.34	0.06	0.3	0.38	30	0.26	0.12
IgG2	0.21	0.1	0.08	0.42	30	0.33	0.08
IgG3	0.25	0.16	0.03	0.71	30	0.68	0.11
IgG4	0.7	0.12	0.51	0.87	30	0.36	0.14
IgE	0.31	0.08	0.22	0.48	30	0.26	0.12

**Table 4:** The mean OD values of IgG class and subclass responses of 30 naturally infected hyadid patients against mice cystic crude fluid antigens.

OD	Mean	Std.Deviation	Minimum	Maximum	Number	Range	Cut-off
IgG	0.35	0.14	0.27	0.43	30	0.5	0.06
IgG2	0.14	0.02	0.12	0.17	30	0.05	0.12
IgG3	0.12	0.004	0.11	0.12	30	0.01	0.16
IgG4	0.29	0.36	0.07	0.97	30	0.9	0.04
IgE	0.12	0.01	0.1	0.14	30	0.04	0.11

**Table 5:** The mean OD values of IgG class and subclass responses of 30 naturally infected hyadid patients against B antigen.

OD	Mean	Std. deviation	Minimum	Maximum	Number	Range	Cut-off
IgG	0.93	0.17	0.81	1.04	30	0.47	0.31
IgG2	1.03	0.17	0.86	1.47	30	0.61	0.29
IgG3	0.77	0.07	0.71	0.94	30	0.23	0.28
IgG4	1.08	0.03	1.04	1.14	30	0.1	0.12
IgE	0.3	0.03	0.26	0.37	30	0.11	0.08

**Table 6:** The mean OD values of IgG class and subclass responses of 30 naturally infected hyadid patients against cattle cystic crude fluid antigens.

OD	Mean	Std. deviation	Minimum	Maximum	Number	Range	Cut-off
IgG	0.32	0.08	0.26	0.37	30	0.33	0.06
IgG2	0.26	0.04	0.21	0.35	30	0.14	0.13
IgG3	0.24	0.15	0.15	0.55	30	0.4	0.07
IgG4	0.82	0.08	0.72	0.94	30	0.22	0.08
IgE	0.16	0.06	0.03	0.3	30	0.28	0.25

**Table 7:** Mean, Standard deviation, confidence interval, and the range of total IgG response of 30 naturally infected human sera against different antigens.

Antigen source	Mean of OD values	Std. deviation	Std. error	95% confidence		Minimum	Maximum
				interval for mean			
				Lower bound	Upper bound		
Human	.5990	.52304	.04299	.5140	.6839	.12	1.99
Sheep	.3402	.19857	.01860	.3033	.3770	.03	.87
Mice	.3489	.31243	.04033	.2682	.4296	.07	.99
Ag B	.9268	.40625	.05745	.8113	1.0422	.26	1.68
Cattle	.3175	.22946	.02762	.2624	.3726	.03	.94

Total	.4912	.42426	.02020	.4515	.5309	.03	1.99

**Table 8:** Correlation between the human IgG responses against different HCF antigens using ANOVA.

OD	Sum of Squares	df	Mean Square	*F*	Sig.
Between groups	17.102	4	4.275	30.019	.01
Within groups	62.097	436	.142		

Total	79.199	440			

**Table 9:** Multivariate analysis of different HCF antigens based on mean OD values of human total IgG responses using post hoc analysis.

(I) Ag	(J) Ag	Mean difference (I-J)	Std. error	Sig.	95% confidence interval	
					Lower bound	Upper bound
Human	Sheep	.25878*	.04703	.000	.1663	.3512
	Mice	.25007*	.05776	.000	.1365	.3636
	B	−.32781*	.06173	.000	−.4491	−.2065
	Cattle	.28147*	.05501	.000	.1734	.3896

Sheep	Human	−.25878*	.04703	.000	−.3512	−.1663
	Mice	−.00871	.06019	.885	−.1270	.1096
	B	−.58658*	.06401	.000	−.7124	−.4608
	Cattle	.02270	.05756	.694	−.0904	.1358

Mice	Human	−.25007*	.05776	.000	−.3636	−.1365
	Sheep	.00871	.06019	.885	−.1096	.1270
	B	−.57788*	.07227	.000	−.7199	−.4358
	Cattle	.03141	.06662	.638	−.0995	.1623

B	Human	.32781*	.06173	.000	.2065	.4491
	Sheep	.58658*	.06401	.000	.4608	.7124
	Mice	.57788*	.07227	.000	.4358	.7199
	Cattle	.60928*	.07009	.000	.4715	.7470

Cattle	Human	−.28147*	.05501	.000	−.3896	−.1734
	Sheep	−.02270	.05756	.694	−.1358	.0904
	Mice	−.03141	.06662	.638	−.1623	.0995
	B	−.60928*	.07009	.000	−.7470	−.4715

* The mean difference is significant at the 0.05 level.

**Table 10:** The mean OD values IgG class, IgG subclass, and IgE of 30 human sample sera to crude HCF antigens of cattle, Ag B, mice, sheep, and human.

	Cattle mean OD	Cut-off	Ag B mean OD	Cut-off	Mice mean OD	Cut-off	Sheep mean OD	Cut-off	Human mean OD	Cut-off
IgG	0.32	0.06	0.93	0.31	0.35	0.06	0.34	0.12	0.59	0.08
IgG2	0.26	0.13	1.03	0.29	0.14	0.12	0.21	0.08	0.34	0.12
IgG3	0.24	0.07	0.77	0.28	0.12	0.16	0.25	0.11	0.16	0.11
IgG4	0.82	0.08	1.08	0.12	0.29	0.04	0.70	0.14	1.58	0.15
IgE	0.16	0.25	0.3	0.08	0.12	0.11	0.31	0.12	0.49	0.06

*P* < 0.01.

**Table 11:** The OD ratio of IgG calass, IgG subclass, and IgE of 30 human sample sera to crude HCF antigens of cattle, Ag B, mice, sheep, and human.

Ig/Ag origin	Cattle	Ag B	Mice	Sheep	Human
IgG	5.3	3	5.8	2.8	7.4
IgG2	2	4.5	1.2	2.6	2.8
IgG3	3.4	2.75	0.75	2.3	1.4
IgG4	10.2	9	7.25	5	10.5
IgE	0.64	3.75	1.1	2.6	8.2

The OD ratio was calculated as mean OD value for each antigen divided by the cut-off of that antigen.
